# ACIST-FFR Study (Assessment of Catheter-Based Interrogation and Standard Techniques for Fractional Flow Reserve Measurement)

**DOI:** 10.1161/CIRCINTERVENTIONS.117.005905

**Published:** 2017-12-13

**Authors:** William F. Fearon, Jeffrey W. Chambers, Arnold H. Seto, Ian J. Sarembock, Ganesh Raveendran, Charlotte Sakarovitch, Lingyao Yang, Manisha Desai, Allen Jeremias, Matthew J. Price

**Affiliations:** From the Stanford University School of Medicine, Department of Medicine, Stanford Cardiovascular Institute, CA (W.F.F., C.S., L.Y., M.D.); Mercy Medical Center, Coon Rapids, MN (J.W.C.); Tibor Rubin VA Medical Center, Long Beach, CA (A.H.S.); Lindner Center for Research and Education, The Christ Hospital, Cincinnati, OH (I.J.S.), University of Minnesota, Minneapolis (G.R.); St. Francis Hospital, Roslyn, NY (A.J.); Cardiovascular Research Foundation, New York, NY (A.J.); and Scripps Clinic, La Jolla, CA (M.J.P.).

**Keywords:** confidence intervals, coronary angiography, coronary artery disease, fractional flow reserve, heart

## Abstract

**Background—:**

Measurement of fractional flow reserve (FFR) to guide coronary revascularization lags despite robust supportive data, partly because of the handling characteristics of traditional coronary pressure wires. An optical pressure-monitoring microcatheter, which can be advanced over a traditional coronary guidewire, facilitates FFR assessment but may underestimate pressure wire–derived FFR.

**Methods and Results—:**

In this prospective, multicenter trial, 169 patients underwent FFR assessment with a pressure wire alone and with a pressure microcatheter over the pressure wire. An independent core laboratory performed quantitative coronary angiography and evaluated all pressure tracings. The primary end point was the bias or difference between the microcatheter FFR and the pressure wire FFR, as assessed by Bland–Altman analysis. The mean difference between the microcatheter and the pressure wire–derived FFR values was −0.022 (95% confidence interval, −0.029 to −0.015). On multivariable analysis, reference vessel diameter (*P*=0.027) and lesion length (*P*=0.044) were independent predictors of bias between the 2 FFR measurements. When the microcatheter FFR was added to this model, it was the only independent predictor of bias (*P*<0.001). The mean FFR value from the microcatheter was significantly lower than from the pressure wire (0.81 versus 0.83; *P*<0.001). In 3% of cases (95% confidence interval, 1.3%–6.7%), there was clinically meaningful diagnostic discordance, with the FFR from the pressure wire >0.80 and that from the microcatheter <0.75. These findings were similar when including all 210 patients with site-reported paired FFR data.

**Conclusions—:**

An optical, pressure-monitoring microcatheter measures lower FFR compared with a pressure wire, but the diagnostic impact appears to be minimal in most cases.

**Clinical Trial Registration—:**

URL: https://www.clinicaltrials.gov. Unique identifier: NCT02577484.

## Abstract 2

Supplemental Digital Content is available in the text.

**WHAT IS KNOWN**

- Measuring fractional flow reserve (FFR) to guide percutaneous coronary interventions improves clinical outcomes.
- FFR can be challenging to measure with a traditional coronary pressure wire because of its handling characteristics, and this may contribute to underutilization of FFR.

**WHAT THE STUDY ADDS**

- This multicenter trial using a core laboratory is the largest comparing FFR measured with a microcatheter to FFR measured with a traditional pressure wire.
- An optical, pressure-monitoring microcatheter measures lower FFR compared with a pressure wire, but the diagnostic impact appears to be minimal in most cases.

Measuring fractional flow reserve (FFR) with a coronary pressure wire has become the reference standard for assessing the functional significance of epicardial coronary artery disease in the cardiac catheterization laboratory.^1^ For many reasons, adoption of FFR measurement into daily practice has lagged behind the data supporting its utilization.^2^ One likely reason is the technical aspect of assessing coronary pressure wire–derived FFR and in particular challenges with manipulating standard coronary pressure wires, hesitancy to pull back the pressure wire sensor to check for pressure drift after having crossed a stenosis, and issues with pressure drift increasing uncertainty about the correct FFR value.

Recently, a microcatheter with an optical pressure sensor was introduced, which can be advanced over any traditional 0.014″ coronary guidewire and maneuvered in and out of the vessel without recrossing the stenosis with the guidewire (Navvus MicroCatheter; ACIST Medical Systems, Inc, Eden Prairie, MN).^3^ By incorporating an optical pressure sensor, the microcatheter system may be less prone to pressure drift seen with the piezo-electric coronary pressure wires. The potential disadvantage of the system is that the microcatheter itself may increase the degree of coronary artery stenosis and lower the measured FFR value.^4^ The influence of the microcatheter on coronary hemodynamics may also depend on lesion and vessel characteristics. To date, these issues have not been investigated in a large, multicenter, prospective fashion across a spectrum of vessel and lesion types and using an independent core laboratory.

## Methods

The goal of the ACIST-FFR study (Assessment of Catheter-Based Interrogation and Standard Techniques for Fractional Flow Reserve Measurement) is to assess the differences, if any, in FFR measurements made by the microcatheter system and a standard coronary pressure wire. The primary end point is the difference in measurement between the microcatheter FFR and the pressure wire FFR, considered a reference standard. Major secondary end points include determining the independent predictors of any bias between the 2 FFR measurements, the diagnostic accuracy of the microcatheter FFR using a threshold of 0.80, device success (defined as a valid FFR reading), presence of clinically significant pressure drift (defined as drift >0.03 in either direction), and the difference in pressure wire FFR with and without the microcatheter across the stenosis.

Eligible patients included those who were 18 years of age or older, who had an intermediate de novo coronary lesion with an operator-assessed reference diameter ≥2.25 mm requiring FFR measurement based on the operator’s clinical judgment. Only 1 stenosis per patient was evaluated. Patients with ST-segment–elevation myocardial infarction or non–ST-segment–elevation myocardial infarction, New York Heart Association class IV heart failure, suspected or visible thrombus, dissection or excessive calcification or tortuosity in the target vessel, or a stenosis in a bypass graft were excluded. All patients provided informed, written consent, and the study was approved by the local ethics committee at each participating center.

After performance of diagnostic angiography and administration of anticoagulation to obtain an activated clotting time >250 s, intracoronary nitroglycerin (100–200 μg) was administered. A standard coronary pressure wire (either Abbott Vascular Inc, Santa Clara, CA or Philips Healthcare, Amsterdam, The Netherlands) was calibrated outside of the body and advanced such that the sensor was positioned at the tip of the guiding catheter where the 2 pressures were equalized and recorded. The wire was then advanced so that the pressure sensor was at least 2 cm beyond the stenosis. The resting distal pressure (P_d_)/proximal pressure (P_a_) was recorded. The pressure microcatheter was then loaded onto the back end of the pressure wire and advanced so that the sensor was positioned at the tip of the guiding catheter where the 2 pressures were equalized and recorded. The microcatheter was then advanced such that the sensor was positioned distal to the target lesion and just a few millimeters proximal to the sensor of the pressure wire. Resting P_d_/P_a_ from both systems was recorded. Intravenous adenosine at 140 μg/kg per minute was administered for at least 2 minutes, and FFR was recorded from both systems. The microcatheter was then pulled back to check for pressure drift, which was recorded. The microcatheter was then removed from the guiding catheter, remaining on the pressure wire, outside of the coronary guide catheter. Intravenous adenosine was restarted at the same dose, and FFR from the pressure wire alone was recorded (with a minimum duration of 2 minutes between adenosine administrations). The pressure wire was then pulled back to check for pressure drift, which was recorded. If either system showed a pressure drift >0.03, the recordings for that system were repeated.

An independent core laboratory (Cardiovascular Research Foundation, New York, NY) evaluated all pressure tracings and performed quantitative coronary angiography on all baseline angiograms for standardized and centralized review. Each subject’s physiology study was assessed for quality based on prespecified criteria that included evaluation of the aortic and coronary pressure signal for waveform distortion or loss, aortic pressure ventricularization, and arrhythmia. Each tracing received a binary decision about adequate quality for inclusion, and P_d_/P_a_ or FFR was calculated independently for each tracing. To evaluate the amount of drift, a final pullback of the pressure device was mandatory. Significant drift was defined as a pressure ratio deviation of >0.03, that is, <0.97 or >1.03, and those cases were removed from the analysis. Subjects were excluded from the primary analysis if the FFR pressure tracing for one or both systems were of inadequate quality, or if drift was significant and the FFR measurement not repeated. All tracings were overread by a physician experienced in physiology measurements to ensure data quality, and feedback was provided to all study sites.

### Statistical Considerations

Comparisons of characteristics of patients included in and excluded from the analytic cohort were performed using χ^2^ tests for qualitative variables and Student *t* test or Mann–Whitney test as appropriate for continuous variables. To assess whether technical issues were differentially present by technique, the percentage of FFR values not recorded because of technical issues was compared between the 2 systems using a McNemar test.

Our primary objective, to compare differences in the microcatheter and pressure wire FFR measurements, was obtained through a Bland–Altman analysis and a 1-sample *t* test. Secondarily, we used a Passing–Bablok regression to characterize the differences in microcatheter and pressure wire through the estimated intercept and slope. For this purpose, the slope and intercept (and 95% confidence interval [CI]) of the linear association between paired FFR measurements by the microcatheter and the pressure wire were characterized. Further prespecified analyses included determining the relationships between differences in measurements, if any, and angiographic characteristics such as vessel interrogated, reference vessel size, stenosis severity, and lesion length as performed through regression techniques. CIs for proportions (sensitivity, specificity, accuracy) were calculated using the Wilson Score method. The mean pressure drift between measurement systems was compared using a paired *t* test. The proportions of cases with clinically significant pressure drift were compared between the 2 measurement systems using the McNemar test. The association between the degree of bias (ie, signed difference between microcatheter and pressure wire) and variables was assessed using linear regression. A multivariable analysis assessing the association between patient and angiographic characteristics with the measurement bias was performed using those variables with a significance level of 0.15 in the univariable analysis. A sensitivity analysis was performed using site-reported FFR measurements rather than the core laboratory–assessed FFR measurements. The sample size for ACIST-FFR was driven principally by the clinical interest in a data set of sufficient size for scientific rigor, and the resulting size of the study was substantially larger than that required by statistical power analysis. A formal statistical evaluation before the study’s initiation was performed for the predicted bias B_C_ at FFR=0.8, with statistical hypotheses defined as follows:


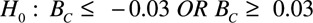



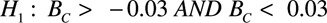


Rejection of the null hypothesis would therefore result in the conclusion that the predicted bias B_C_ was within the acceptable range. A postulated standard deviation of 0.056 for the paired difference of FFR measurements (ie, the bias) B_C_ was computed from the previous ACCESS-NZ study (Assessment of Coronary Fractional Flow Reserve Using a Monorail Pressure Catheter), using the ACCESS-NZ standard deviation of 0.11 for FFR measurement and an observed correlation of 0.87 between the pressure wire and microcatheter FFR measurements. This results in a statistical sample size of just 32 subjects. The actual evaluable sample size of 169 was therefore sufficient to assess this objective from a statistical standpoint. Hypothesis tests were conducted using 2-sided tests at a 0.05 level of significance. Statistical analyses were performed with R software^5^ at Stanford University, independently from the Sponsor for all analyses with the exception of those corresponding to the device success. The data, analytic methods, and study materials will not be made available to other researchers for purposes of reproducing the results or replicating the procedure.

## Results

**Figure 1.**
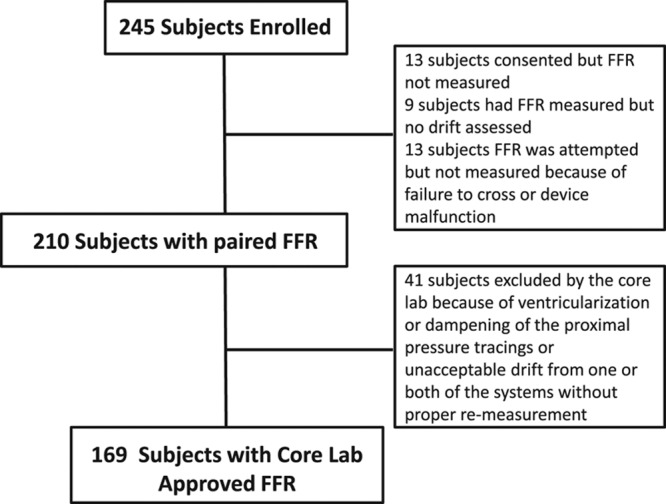
Flowchart depicting patients included and excluded in the study. FFR indicates fractional flow reserve.

A total of 245 patients were enrolled from 11 participating centers. The study flowchart is outlined in Figure 1. Thirteen subjects were consented, but FFR was never ultimately measured. In 9 subjects, FFR was measured, but there was no assessment of drift. In 13 patients, FFR measurement was attempted but not completed because of inability to cross the lesion with the microcatheter or because of device (microcatheter or wire) malfunction. The pressure wire crossed the target lesion successfully in all cases; in 8 cases (3%), the microcatheter did not cross. In 210 patients, successful FFR measurement with both systems with adequate assessment of pressure drift was achieved. A total of 41 patients were further excluded by the core laboratory because of concerns about the pressure tracings (dampening or ventricularization of the proximal pressure) or pressure drift from one or both systems. Thus, the primary analysis included the 169 patients who had FFR successfully measured from both systems without clinically significant drift and with acceptable tracings according to the core laboratory. In 115 cases, the pressure wire FFR was measured with the Abbott Vascular (previously St. Jude Medical) pressure system, and in 54 cases, it was measured with the Philips Healthcare (previously Volcano) pressure system.

The patient characteristics are listed in Table 1, and the angiographic characteristics are listed in Table 2. The mean reference vessel diameter was 2.8±0.5 mm (30% of cases were <2.5 mm), the mean lesion length was 15.3±8 mm, and the mean diameter stenosis was 47±9%. Patients excluded from the analysis (n=76) were comparable to those included in the analysis (n=169) for the majority of the baseline characteristics. Those excluded, however, were more likely to have dyslipidemia (87% versus 75%; *P*=0.04) and a different distribution of angina severity (Table I in the Data Supplement). The angiographic characteristics of the 2 groups were similar (Table II in the Data Supplement).

**Table 1.**
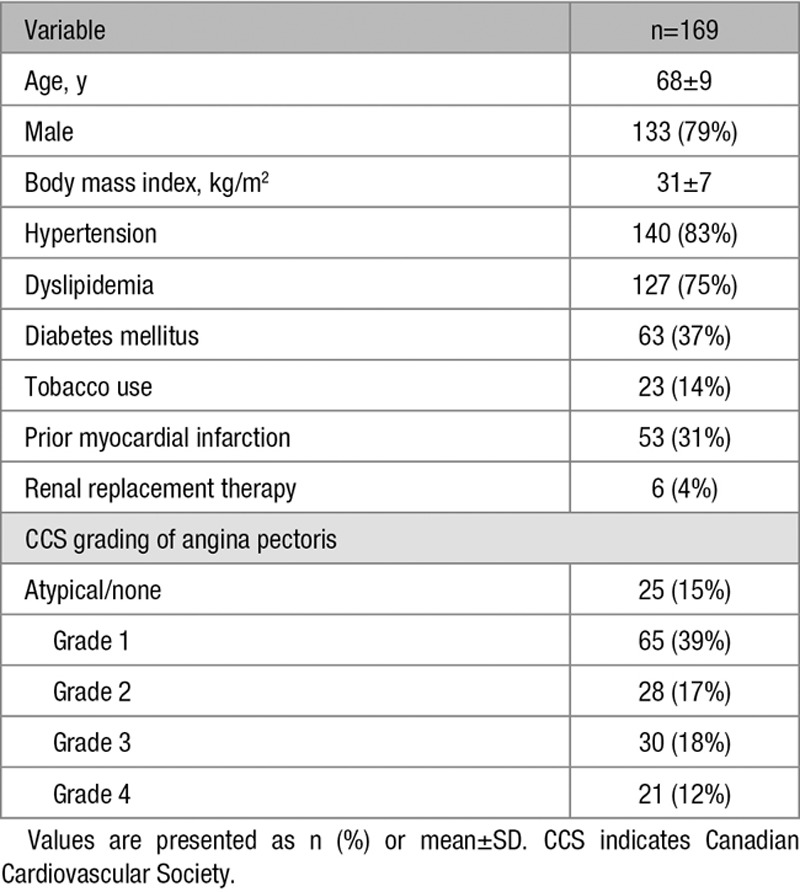
Clinical Characteristics

**Table 2.**
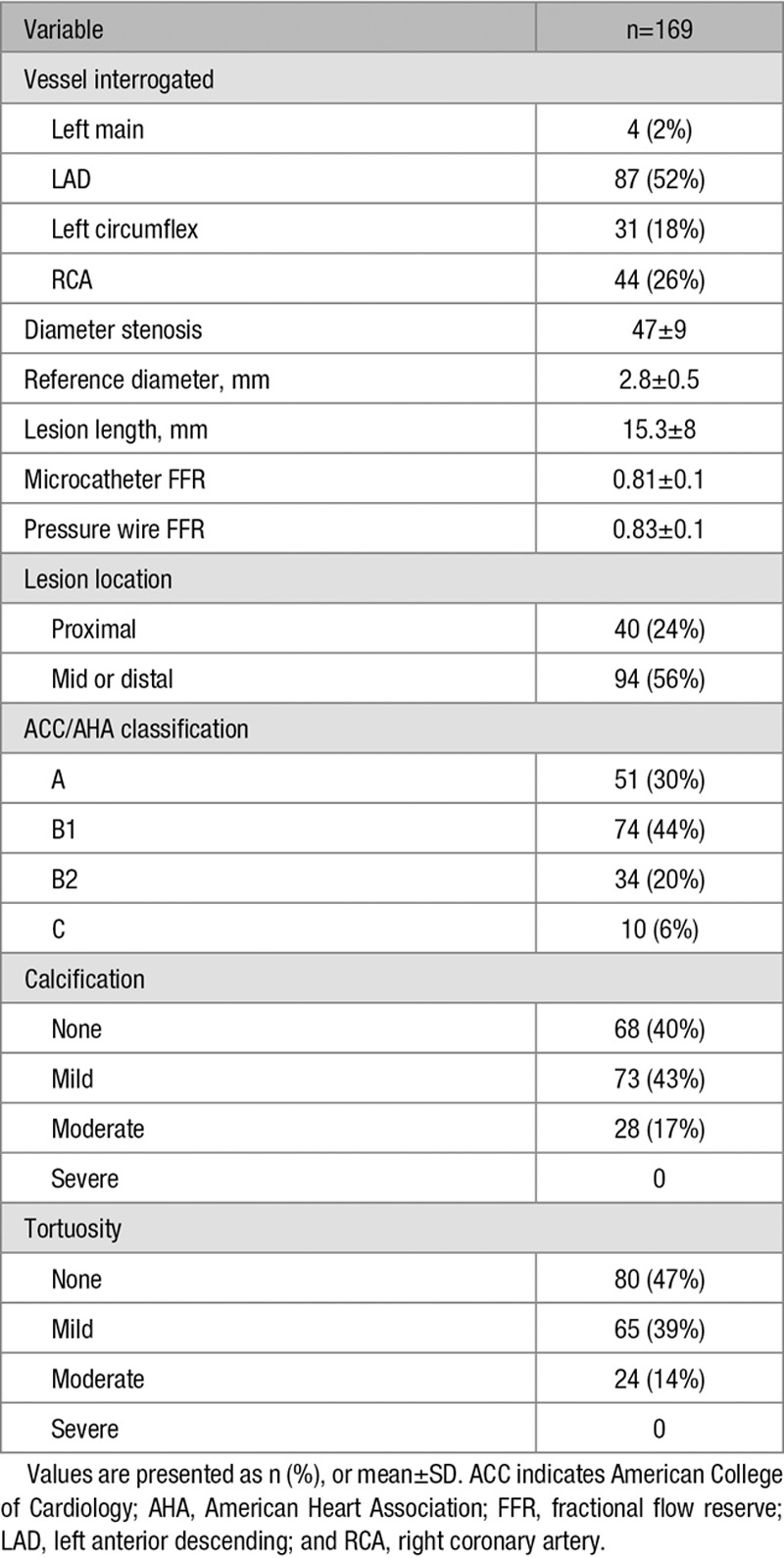
Angiographic Characteristics

**Figure 2.**
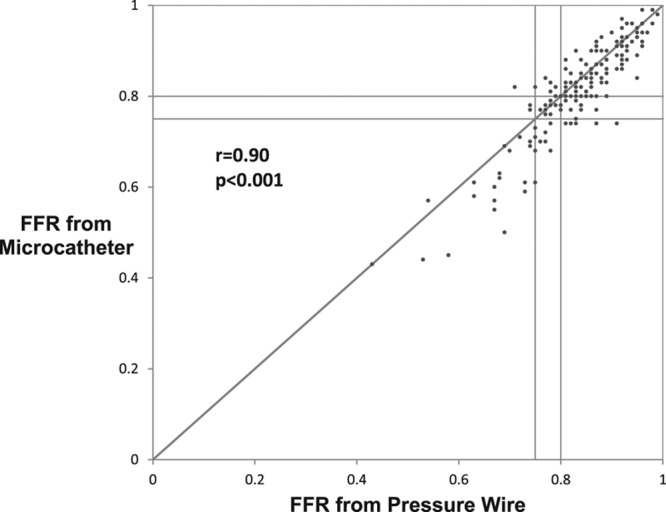
Correlation between the microcatheter and pressure wire fractional flow reserve (FFR) measurements.

**Figure 3.**
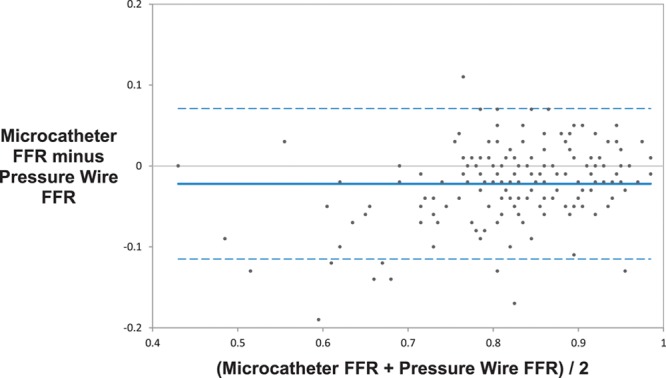
Bland–Altman plot comparing fractional flow reserve (FFR) measurements from the microcatheter to those from the pressure wire.

**Figure 4.**
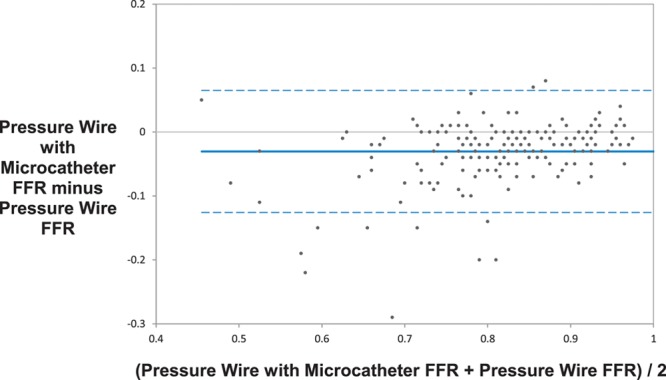
Bland–Altman plot comparing fractional flow reserve (FFR) measurements from the pressure wire with and without the microcatheter on top of the wire.

**Figure 5.**
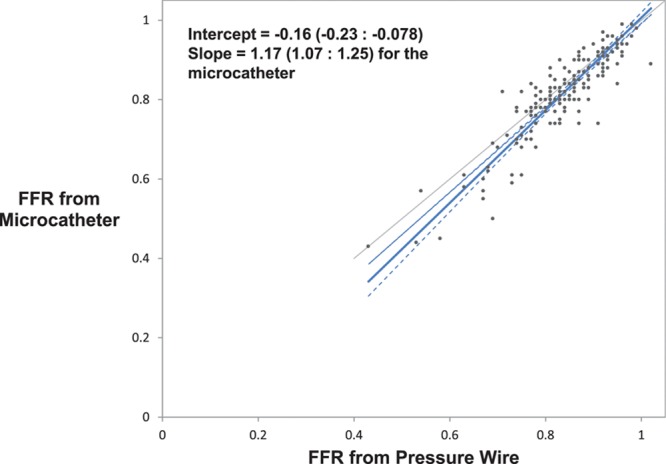
The slope and intercept (and 95% CI [confidence interval]) of the Passing–Bablok regression between paired fractional flow reserve (FFR) measurements by the microcatheter and pressure wire.

**Figure 6.**
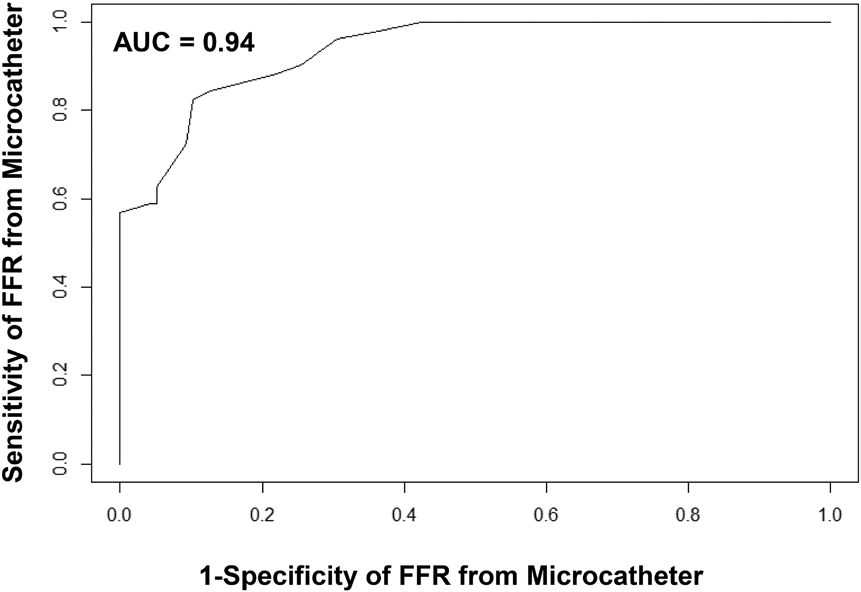
Receiver operator characteristic curve comparing fractional flow reserve (FFR) measurement with the microcatheter to FFR measurement with the pressure wire. AUC indicates area under the curve.

Device success, defined as a successful FFR measurement, was significantly higher with the pressure wire compared with the microcatheter (98.7% [232/235] versus 95.0% [224/234], paired data only; *P*=0.021). Subjects enrolled but not completing the protocol were not included in this rate. The mean microcatheter FFR was significantly lower than the mean pressure wire FFR (0.81 versus 0.83; *P*<0.001). There was a strong correlation between the microcatheter and pressure wire FFR measurements (*r*=0.90; *P*<0.001; Figure 2). The primary end point, the average difference between the microcatheter FFR and the pressure wire FFR, was −0.022 (95% CI, −0.029 to −0.015) as assessed by Bland–Altman analysis (Figure 3). Similarly, the mean FFR measured with the pressure wire alone was significantly higher than the mean FFR measured with the same pressure wire, but with the microcatheter across the stenosis (0.84 versus 0.81; *P*<0.001); the bias between these 2 measurements as assessed by Bland–Altman analysis was −0.032 (95% CI, −0.038 to −0.025; Figure 4). The slope and intercept (and 95% CI) of the Passing–Bablok regression between paired FFR measurements by the microcatheter and pressure wire were 1.17 (95% CI, 1.07–1.25) and −0.16 (95% CI, −0.23 to −0.08), respectively (Figure 5). At a pressure wire FFR=0.80, the Passing–Bablok shows a difference of −0.027 (95% CI, −0.035 to −0.016). Using a cutoff value of ≤0.80 measured with the pressure wire, the sensitivity, specificity, and diagnostic accuracy of the microcatheter-derived FFR were 88% (95% CI, 76%–96%), 78% (95% CI, 69%–85%), and 81% (95% CI, 75%–87%), respectively. Using a cutoff value of ≤0.78 measured with the pressure wire, the sensitivity, specificity, and diagnostic accuracy of the microcatheter-derived FFR improved to 82% (95% CI, 69%–92%), 90% (95% CI, 83%–95%), and 88% (95% CI, 82%–92%), respectively. The area under the curve of the receiver operator characteristic curve comparing microcatheter-derived FFR to pressure wire–derived FFR was 0.94 (Figure 6). The FFR values with the microcatheter and pressure wire differed by >0.05 in 47 (28%) cases. However, there were only 5 cases (3.0%; 95% CI, 1.3%–6.7%) in which the pressure wire FFR was >0.80, and the microcatheter FFR value was <0.75 (the accepted grey zone for pressure wire FFR).

### Drift

The mean pressure drift was not different between the pressure wire and the microcatheter (0.015±0.017 versus 0.014±0.012; *P*=0.66; the rate of clinically significant pressure drift with the pressure wire in paired drift data was 7.4% (15/203) compared with 3.5% (7/203) for the microcatheter (*P*=0.10).

### Sensitivity Analysis

There was a strong correlation between the site-reported pressure wire FFR values and the core laboratory–assessed pressure wire FFR values (*r*=0.99; *P*<0.001; n=216); the same was true when comparing the microcatheter FFR values reported by the sites and the core laboratory (*r*=0.96; *P*<0.001; n=213; Figures I and II in the Data Supplement). The mean difference between the microcatheter FFR and the pressure wire FFR from the 221 site-reported values as assessed by Bland–Altman analysis was −0.025 (95% CI, −0.034 to −0.016).

### Predictors of Bias Between FFR Methods

On univariable analysis, the magnitude of bias between the 2 FFR systems tended to be associated with reference vessel diameter (*P*=0.058) and with lesion length (*P*=0.10). Lesion location (for proximal or nonproximal location, *P*=0.58) and interrogated vessel (for left anterior descending versus non–left anterior descending, *P*=0.57) were not significant predictors. Furthermore, the difference between the 2 systems was not associated with clinical characteristics listed in Table 1. On multivariable analysis, both reference vessel diameter (*P*=0.027) and lesion length (*P*=0.044) were significant independent predictors of the difference between the 2 systems. When the FFR measured from the microcatheter was included in the analysis, it was a significant univariable predictor of the difference between the 2 systems (*P*<0.001) and it was the only significant independent predictor of the difference (*P*<0.001), with reference vessel diameter and lesion length no longer significant predictors.

## Discussion

The main finding of this study is that a pressure-monitoring microcatheter system provides a lower FFR value compared with a traditional coronary pressure wire, with an average bias of −0.02. Importantly, this finding is unchanged whether all site-reported values are included in the analysis or only the core laboratory–accepted FFR values are included. The magnitude of the difference between the FFR measured with the microcatheter when compared with the pressure wire alone is related to the reference vessel diameter and the length of the lesion being interrogated. From a physiological standpoint, it makes sense that the addition of the microcatheter would have a greater impact on flow across the stenosis, leading to a larger gradient and lower FFR compared with in the absence of the microcatheter in the setting of smaller vessels and longer lesions. Although one might expect the addition of the microcatheter across a proximal lesion or across a left anterior descending lesion to be associated with more bias between the 2 techniques because of the greater increase in flow across lesions in these locations, this was not the case.

When including the FFR from the microcatheter in the multivariable model, the FFR from the microcatheter was the only significant predictor, independent of vessel and lesion characteristics. The more physiologically severe the lesion as assessed by FFR from the microcatheter, the greater the difference between the FFR from the microcatheter and the pressure wire. However, in most of these cases, both FFR values were below the ischemic threshold and, therefore, the observed bias did not impact clinical decision-making. In only 3% of cases, did we find an FFR value for the pressure wire alone above the ischemic threshold of 0.80, whereas the FFR value for the microcatheter was below the lower end of the FFR gray zone of 0.75.

To date, 2 studies have been published comparing FFR measured with the pressure microcatheter to FFR measured with a pressure wire. In the ACCESS-NZ study, Menon et al^3^ compared FFR measured with a pressure wire alone to FFR measured with the pressure microcatheter in 50 patients in target lesions with reference vessel diameter ≥2.5 mm. They demonstrated a strong correlation between the 2 devices (*r*=0.87) with a mean microcatheter FFR value of 0.79 and a mean pressure wire FFR value of 0.81. Furthermore, they found that the microcatheter FFR was concordant with the pressure wire FFR with respect to clinical decision-making at a cutoff value of <0.80 after accounting for the measurement variability of the pressure wire (=±0.045). There was significantly greater drift with the pressure wire system compared with the microcatheter, although a few dramatic outliers in the pressure wire group might have influenced this finding.

In the second published study comparing these 2 techniques, Wijntjens et al^4^ measured FFR and coronary flow velocity in 28 patients with a dual pressure/Doppler sensor tip guidewire with and without the pressure microcatheter. They observed that FFR was significantly lower in the presence of the microcatheter compared with in its absence (0.82 versus 0.86; *P*<0.001). The mean bias based on Bland–Altman analysis was −0.033 (limits of agreement: −0.09 to 0.03). Passing–Bablok analysis revealed a significant constant and significant proportional difference between the 2 methods. From the Doppler measurements, they also attributed a significant increase in stenosis resistance to introduction of the microcatheter. There was a trend toward less pressure drift with the microcatheter when compared with the pressure/Doppler wire.

The current study confirms and expands on these previous findings in a much larger cohort and in a multicenter trial with core laboratory analysis. Overall, the microcatheter introduces a modest pressure offset which tends to be greater in smaller vessels and longer lesions, resulting in a lower FFR value when compared with the pressure wire alone. Passing–Bablok analysis confirms a modest, constant, and proportional difference between methods. Unique to our study, the large number of patients and broad angiographic entry criteria across a spectrum of lesion and vessel characteristics, including 30% of patients with vessel diameter <2.5 mm, provides new information on which patient and lesion characteristics contribute most to the observed bias with the microcatheter.

The clinical implications of our findings are that the impact of the microcatheter is negligible at very high FFR values, modest near the 0.80 cut point, and significant at very low FFR values. Some operators will defer revascularization if the pressure wire FFR is >0.80 and perform revascularization if it is <0.75. When the pressure wire FFR value falls in between these values, clinical judgment on the benefit of revascularization is incorporated into the final decision. Moreover, because the FFR measured from the microcatheter was the only independent predictor of bias between the 2 FFR techniques, it takes into account lesion and vessel characteristics, such as vessel size and lesion length. For example, if the FFR from the microcatheter is 0.82 in a smaller vessel with a longer lesion, the operator does not need to be concerned that a larger degree of bias will be present compared with a similar FFR measured in a larger vessel with a more focal lesion.

A potential advantage of the microcatheter system is the utilization of an optical pressure sensor, which may be prone to less pressure drift.^6^ Indeed, previous studies have found less drift with the microcatheter.^3^ In this study, there was less clinically significant drift with the microcatheter, but this difference did not reach statistical significance. Other unique features of the microcatheter FFR system include the ability to use it with any traditional 0.014″ coronary guidewire and the ability to maintain guidewire position throughout the procedure. A potential disadvantage of the microcatheter system is the inability to cross some lesions that might be crossed by a pressure wire. Although the microcatheter could not be advanced over the pressure wire past the target lesion in 8 cases (3.2%), this may not occur as frequently when the microcatheter is advanced over a workhorse or other type of coronary guidewire. In addition, this trial tested a first-generation microcatheter. A second-generation microcatheter with reduced lesion entry profile is now currently commercially available, but was not available during the enrollment phase of this study. The improvements in the tip of the second-generation microcatheter may lead to improved ability to cross narrowed, tortuous, and calcified vessels. Although not tested specifically in this study, one would expect the difference in resting pressure wire indices to be less between the 2 FFR techniques. This is the subject of future study.

Limitations of this study include the significant minority of FFR measurements which were excluded from the final analysis by the core laboratory; however, when including all measurements based on site-reported values, the findings were similar. The lack of randomizing the order of measurements between the pressure wire and the microcatheter FFR is another possible limitation, as the second hyperemic response to adenosine might differ from the first. However, previous studies have demonstrated excellent reproducibility of FFR measurements on repeated testing.^7^ Only intravenous adenosine was used in this study because it is considered the reference standard for hyperemia. Results with intracoronary adenosine might differ, if less hyperemic effect is achieved. This study did not include Doppler flow measurements to provide further support for the mechanistic explanation for the findings; however, this was done previously.^4^ This study did not address clinical outcomes based on using microcatheter-derived FFR to guide percutaneous coronary intervention. Finally, this study included 2 different commercially available pressure wire systems, which may have introduced variability to the pressure wire FFR measurements.

## Conclusions

In summary, a pressure microcatheter measures lower FFR values compared with a traditional pressure wire, particularly across more physiologically severe lesions, as assessed by the microcatheter FFR. The clinical impact of this difference appears to be minimal in most cases.

## Sources of Funding

This work was supported by ACIST Medical Systems, Inc, Eden Prairie, MN.

## Disclosures

Dr Fearon receives research support from ACIST Medical Systems, Abbott, Medtronic, and CathWorks and has a consulting relationship with HeartFlow. Dr Seto receives research support from ACIST Medical Systems and speaking fees from ACIST Medical Systems and Volcano/Philips. Dr Jeremias is a consultant for Abbott Vascular and Philips and on the Speaker’s Bureau of Medtronic. Dr Price is a consultant for AstraZeneca, ACIST Medical, Boston Scientific, Medtronic, St. Jude Medical, and The Medicines Company; and he receives speaker’s fees from AstraZeneca, Abbott Vascular, Medtronic, St. Jude Medical, Terumo, Chiesi USA, and The Medicines Company. The other authors report no conflicts.

## Supplementary Material

## References

[1] Fearon WF (2014). Percutaneous coronary intervention should be guided by fractional flow reserve measurement.. Circulation.

[2] Pothineni NV, Shah NN, Rochlani Y, Nairooz R, Raina S, Leesar MA, Uretsky BF, Hakeem A (2016). U.S. trends in inpatient utilization of fractional flow reserve and percutaneous coronary intervention.. J Am Coll Cardiol.

[3] Menon M, Jaffe W, Watson T, Webster M (2015). Assessment of coronary fractional flow reserve using a monorail pressure catheter: the first-in-human ACCESS-NZ trial.. EuroIntervention.

[4] Wijntjens GW, van de Hoef TP, Kraak RP, Beijk MA, Sjauw KD, Vis MM, Madera Cambero MI, Brinckman SL, Plomp J, Baan J, Koch KT, Wykrzykowska JJ, Henriques JP, de Winter RJ, Piek JJ (2016). The IMPACT Study (Influence of Sensor-Equipped Microcatheters on Coronary Hemodynamics and the Accuracy of Physiological Indices of Functional Stenosis Severity).. Circ Cardiovasc Interv.

[5] R Core Team (2015). R: A Language and Environment for Statistical Computing.

[6] Diletti R, Van Mieghem NM, Valgimigli M, Karanasos A, Everaert BR, Daemen J, van Geuns RJ, de Jaegere PP, Zijlstra F, Regar E (2015). Rapid exchange ultra-thin microcatheter using fibre-optic sensing technology for measurement of intracoronary fractional flow reserve.. EuroIntervention.

[7] Johnson NP, Jeremias A, Zimmermann FM, Adjedj J, Witt N, Hennigan B, Koo BK, Maehara A, Matsumura M, Barbato E, Esposito G, Trimarco B, Rioufol G, Park SJ, Yang HM, Baptista SB, Chrysant GS, Leone AM, Berry C, De Bruyne B, Gould KL, Kirkeeide RL, Oldroyd KG, Pijls NHJ, Fearon WF (2016). Continuum of vasodilator stress from rest to contrast medium to adenosine hyperemia for fractional flow reserve assessment.. JACC Cardiovasc Interv.

