# Supplementary material for: ACIST-FFR Study (Assessment of Catheter-Based Interrogation and Standard Techniques for Fractional Flow Reserve Measurement)
Source: Circ Cardiovasc Interv. 2017 Dec 13;10(12):e005905. doi: 10.1161/CIRCINTERVENTIONS.117.005905 (PMC5753822; doi:10.1161/CIRCINTERVENTIONS.117.005905)

SUPPLEMENTAL MATERIAL

Supplemental Table 1. Clinical characteristics comparison between included and excluded cases

**Variable**	**n = 169**	**n = 76**
Age, years	68 ±9	67 ±11
Male	133 (79%)	52 (68%)
Body Mass Index, kg/m^2	31 ±7	30 ±6
Hypertension	140 (83%)	64 (84%)
Dyslipidemia	127 (75%)	66 (87%)
Diabetes	63 (37%)	38 (50%)
Tobacco Use	23 (14%)	9 (12%)
Prior Myocardial Infarction	53 (31%)	20 (26%)
Renal Replacement Therapy	6 (4%)	4 (5%)
CCS Grading of Angina Pectoris		
Atypical/None	25 (15%)	18 (24%)
Grade 1	65 (39%)	17 (22%)
Grade 2	28 (17%)	17 (22%)
Grade 3	30 (18%)	22 (29%)
Grade 4	21 (12%)	2 (3%)

Values are presented as n (%) or mean ± std. CCS indicates Canadian Cardiovascular Society.

Supplemental Table 2. Angiographic characteristics comparison between included and excluded cases.

Variable	n = 169	n = 76
Vessel Interrogated		
Left Main	4 (2%)	0 (0%)
LAD	87 (52%)	42 (55%)
Left Circumflex	31 (18%)	18 (23%)
RCA	44 (26%)	16 (21%)
Diameter Stenosis	47 ±9	47 ±9
Reference Diameter (mm)	2.8 ±0.5	2.8 ±0.6
Minimum Lumen Diameter (mm)	1.48 ±0.37	1.49 ±0.42
Lesion Length (mm)	15.3 ±8	13.4 ±8
Microcatheter FFR	0.81±0.1	0.78 ±0.1
Pressure Wire FFR	0.83±0.1	0.82 ±0.08
Lesion Location		
Proximal	40 (24%)	20 (26%)
Mid or Distal	94 (56%)	46 (60%)
ACC/AHA Classification		
A	51 (30%)	19 (25%)
B1	74 (44%)	36 (47%)
B2	34 (20%)	13 (17%)
C	10 (6%)	8 (1%)
Calcification		
None	68 (40%)	31 (41%)
Mild	73 (43%)	31 (41%)
Moderate	28 (17%)	14 (18%)
Severe	0	0
Tortuosity		
None	80 (47%)	34 (44%)
Mild	65 (39%)	29 (38%)
Moderate	24 (14%)	13 (17%)
Severe	0	0

Values are presented as n (%), or mean ± std . LAD indicates left anterior descending; RCA indicates right coronary artery; ACC indicates American College of Cardiology and AHA American Heart Association.

Supplemental Figure Legend:

Supplemental Figure 1. Correlation between FFR values from the pressure wire reported by the sites and from the pressure wire reported by the core laboratory.

Supplemental Figure 2. Correlation between FFR values from the microcatheter reported by the sites and from the microcatheter reported by the core laboratory.

Supplemental Figure 1.

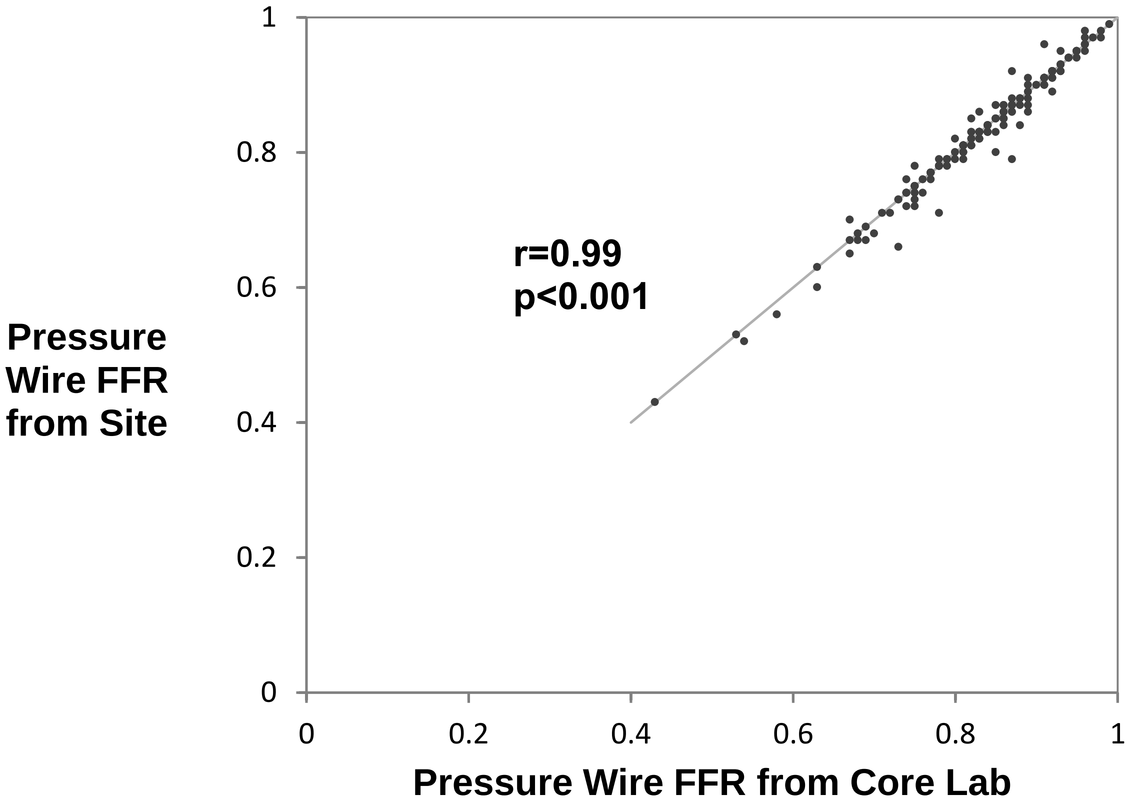

Supplemental Figure 2.

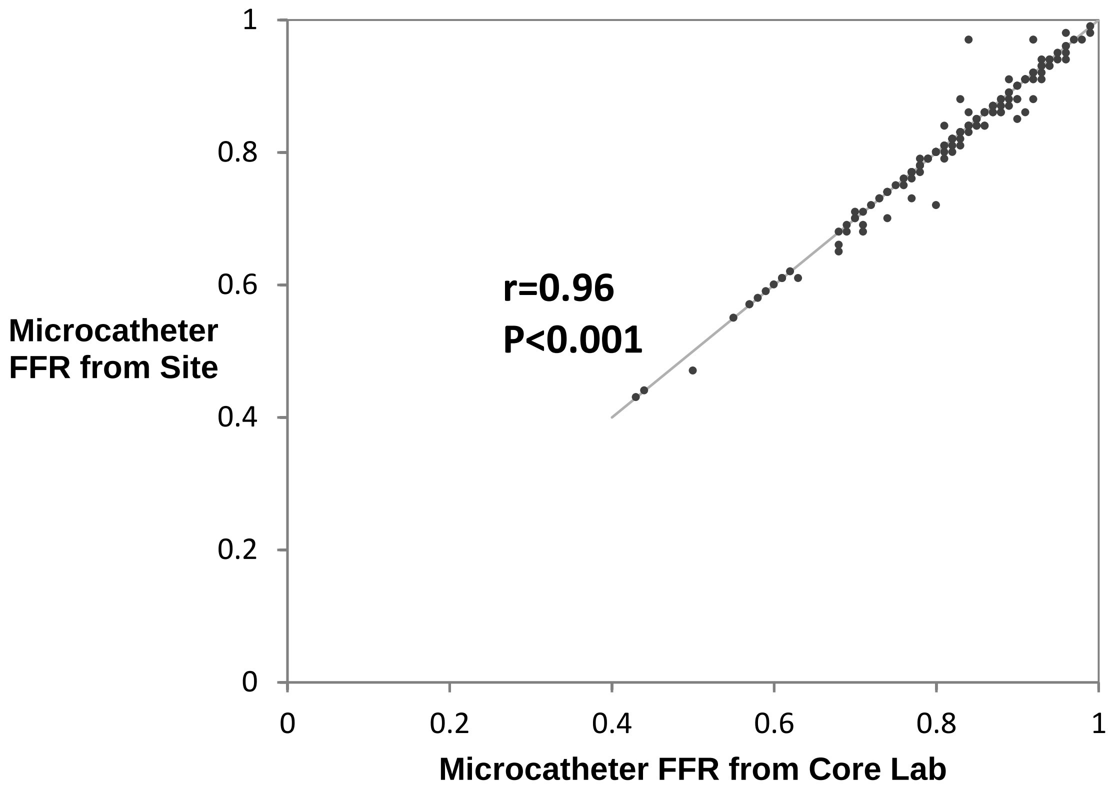

Supplement: Supplementary file 1 [file hcv-10-e005905-s001.pdf]
